# Changes in molecular characteristics and antimicrobial resistance of invasive *Staphylococcus aureus* infection strains isolated from children in Kunming, China during the COVID-19 epidemic

**DOI:** 10.3389/fmicb.2022.944078

**Published:** 2022-08-11

**Authors:** Mingbiao Ma, Lvyan Tao, Xinyue Li, Yanqi Liang, Jue Li, Haiping Wang, Hongchao Jiang, Jing Dong, Dingrui Han, Tingyi Du

**Affiliations:** ^1^Department of Clinical Laboratory, Kunming Children’s Hospital, Kunming, China; ^2^Yunnan Key Laboratory of Children’s Major Disease Research, Kunming Children’s Hospital, Kunming, China; ^3^Yunnan Province Clinical Research Center for Children’s Health and Disease, Kunming Children’s Hospital, Kunming, China; ^4^Yunnan Institute of Pediatrics, Kunming Children’s Hospital, Kunming, China; ^5^Department of Laboratory, Chuxiong Higher College of Medicine, Chuxiong, China

**Keywords:** *Staphylococcus aureus*, children, invasive infection, COVID-19, molecular characteristic, antimicrobial

## Abstract

Invasive *Staphylococcus aureus* (*S. aureus*) infection is associated with high rates of mortality in children. No studies have been reported on invasive *S. aureus* infection among children in Kunming, China, and it remains unknown whether the COVID-19 epidemic has affected *S. aureus* prevalence in this region. Thus, this study investigated the changes in molecular characteristics and antimicrobial resistance of invasive *S. aureus* strains isolated from children in Kunming during 2019–2021. In total, 66 invasive *S. aureus* strains isolated from children were typed by multilocus sequence typing (MLST), *spa*, and Staphylococcal cassette chromosome mec (SCC*mec*), and antimicrobial resistance and virulence genes were analyzed. A total of 19 ST types, 31 *spa* types and 3 SCC*mec* types were identified. Thirty nine (59.09%) strains were methicillin-sensitive *S. aureus* (MSSA) and 27 (40.91%) strains were methicillin-resistant *S. aureus* (MRSA). The most common molecular type was ST22-t309 (22.73%, 15/66), followed by ST59-t437 (13.64%, 9/66). In 2019 and 2021, the dominant molecular type was ST22-t309, while in 2020, it was ST59-t437. After 2019, the dominant molecular type of MRSA changed from ST338-t437 to ST59-t437. All strains were susceptible to tigecycline, ciprofloxacin, moxifloxacin, vancomycin, quinopudine-dafoputin, linezolid, levofloxacin, and rifampicin. From 2019 to 2021, the resistance to penicillin and sulfamethoxazole initially decreased and then increased, a trend that contrasted with the observed resistance to oxacillin, cefoxitin, erythromycin, clindamycin, and tetracycline. Sixteen antimicrobial resistance profiles were identified, with penicillin-tetracycline-erythromycin-clindamycin-oxacillin-cefoxitin being the most common, and the antimicrobial resistance profiles varied by year. The carrier rates of virulence genes, *ica*A, *ica*D, *hla*, *fnb*A, *fnb*B, *clf*A, *clf*B, and *cna* were 100.00%. Furthermore, *sak*, *pvl*, *ica*C, *ica*R, *fib*, *lip*, *hlb*, *hysA*, *sea*, *seb*, and *tsst*-1 had carrier rates of 96.97, 92.42, 87.88, 69.70, 84.85, 62.12, 56.06, 50, 37.87, 30.30, and 7.58%, respectively. Since COVID-19 epidemic, the annual number of invasive *S. aureus* strains isolated from children in Kunming remained stable, but the molecular characteristics and antimicrobial resistance profiles of prevalent *S. aureus* strains have changed significantly. Thus, COVID-19 prevention and control should be supplemented by surveillance of common clinical pathogens, particularly vigilance against the prevalence of multidrug-resistant and high-virulence strains.

## Introduction

*Staphylococcus aureus* (*S. aureus*) has emerged as one of the most common bacteria in hospital and community-acquired infections, causing a wide range of infections including skin and soft tissue infections, cellulitis, and arthritis. It can also induce invasive infections involving deep tissues or organs, such as bacteremia, meningitis, osteomyelitis, and septicemia (Magill et al., 2018). *S. aureus* is more likely to cause severe invasive infections in children, whose immune systems, mucosa, and biological barriers are not yet fully developed (Ondusko and Nolt, 2018).

The incidence rate of invasive *S. aureus* infection has obvious regional and temporal differences. Williamson et al. (2014) found that the incidence rate of invasive *S. aureus* infection was approximately 52/100,000 among children in New Zealand. In the United States, the incidence rate of invasive community-associated methicillin-resistant *S. aureus* (CA-MRSA) infection among children rose from 1.10/100,000 in 2005 to 1.70/100,000 in 2010, with an annual growth rate of 10.20% (Iwamoto et al., 2013). The incidence rate of invasive MRSA infection in Chinese children increased from 3.75/10,000 in 2006 to 8.90/10,000 in 2011, with the incidence of invasive CA-MRSA infection increasing from zero to 2.43/10,000 (Qiao et al., 2013). These findings suggest that the incidence rate of invasive *S. aureus* infection in Chinese children is higher than that observed in developed countries.

More worrisome is the fact that invasive *S. aureus* infection in children is often accompanied by severe clinical signs and symptoms, which is even life-threatening. The mortality rate of children with invasive *S. aureus* infection has increased to 14.00% in the United States, of whom 30.00% die of sepsis despite appropriate treatment (Iwamoto et al., 2013). Li et al. (2015) showed that of 56 Chinese children with invasive *S. aureus* infection, two died, 35.72% had dyspnea, 28.57% were admitted to the ICU, and 37.5% had severe complications such as respiratory failure. These findings indicate that invasive *S. aureus* infection is a serious threat to the health and life of children and poses a significant challenge to clinical treatment.

The distribution characteristics of *S. aureus* differ considerably by geographic region. Studies indicate that ST8 and ST121 are the dominant *S. aureus* strains responsible for invasive infection in the United States, ST59-t437 and ST398-t571 in China, ST8-t190 in Australia, ST8-t008 and ST5-t777 in France, ST22-t032 in Germany, and ST22-t032 in the United Kingdom (Grundmann et al., 2010; O’Hara et al., 2012; Qiao et al., 2013). The molecular characteristics of prevalent *S. aureus* strains also vary over time. Wang et al. (2021a) found that the *S. aureus* strain responsible for bloodstream infection was ST398-t571 from 2016 to 2017 and ST59-t437 from 2018 to 2020 in Shandong Province, China. Thus, it is important to continuously monitor the prevalence of S. aureus in various regions. Unfortunately, no studies on invasive *S. aureus* infection among children in Kunming have been reported yet.

Since December 2019, COVID-19 has rapidly spread worldwide, and a variety of prevention and control measures have been employed in different regions and countries. The lifestyle of Chinese people has changed greatly since the COVID-19 pandemic, including lower population movement between different regions and provinces, wearing a mask, home quarantine, and COVID-19 vaccination. A study indicated that the positivity rates of viruses and atypical pathogens responsible for acute respiratory tract infection (ARI) in Chinese children have declined significantly since the COVID-19 pandemic and that the pathogenic spectrum has also changed (Wang et al., 2021b). Therefore, it is also worth considering and studying whether these changes have also affected the prevalence of common clinical bacterial pathogens.

To reveal the impact of the COVID-19 pandemic, this study aims to investigate the changes in molecular characteristics and antimicrobial resistance of invasive *S. aureus* strains isolated from children in Kunming during 2019–2021.

## Materials and methods

### Strain collection and identification

Invasive *S. aureus* infection is an infection in which *S. aureus* is isolated from a normally sterile part of the human body. Samples collected repeatedly from the same body part of the same patient should be eliminated. From January 2019 to December 2021, 66 invasive *S. aureus* strains (22, 24, and 20 in 2019, 2020, and 2021, respectively) were isolated from children < 18 years of age who were treated at Kunming Children’s Hospital, also called Yunnan Children’s Medical Center. This medical center is an affiliated hospital of Kunming Medical University, the largest tertiary hospital for children in Yunnan Province, and is responsible for the diagnosis and treatment of all refractory and critically ill children in the province.

Clinical samples include sterile body fluid specimens such as blood, cerebrospinal fluid, pleural fluid, bone marrow, and alveolar lavage fluid. The samples were inoculated onto blood agar plates and cultured in 5% CO_2_ at 37°C for 24 h. The strain colonies were subjected to morphological observation, Gram staining and microscopic examination, automatic bacterial identification using VITEK-2 Compact. MRSA was preliminarily determined based on a positive result of cefoxitin screening and confirmed by the presence of the *mec*A gene using PCR (Stegger et al., 2012).

### Multilocus sequence typing

All stains were subjected to molecular typing by Multilocus sequence typing (MLST). Seven housekeeping genes (*arcC*, *aroE*, *glpF*, *gmk*, *pta*, *tpi*, and y*qiL*) of MLST primer sequences and reaction conditions amplified by PCR were obtained from the MLST website.^1^ The sequences of the PCR products were compared to those of the existing alleles available from the MLST website, and the allelic number (sequence type, ST) was determined for each sequence.

### *Spa* typing

All strains were typed by the *spa* typing according to published protocols, by amplifying the *spa* X region (Koreen et al., 2004). The PCR products were sequenced. Repeats and *spa* types were assigned using the *spa* database.^2^ The strains that could not be classified as any known *spa* type were defined as non-typable (NT).

### Staphylococcal cassette chromosome mec typing

The SCC*mec* classification of all MRSA strains was performed as previously described (Zhang et al., 2005).

### VITEK 2 Compact automated antimicrobial susceptibility test

The antimicrobial susceptibility test (AST) was performed according to the VITEK 2 Compact Automatic Bacterial Identification and Antimicrobial Susceptibility Analyzer user manual. In brief, a 0.5 MCF bacterial suspension was prepared. The instrument automatically adds the bacteria suspension to the Gram-positive bacteria AST card GP-639. After 18–24 h incubation, the Advanced Expert System™ system analyzes the susceptibility of bacteria to various antibacterial drugs. The interpretation of AST results was based on Clinical and Laboratory Standards Institute (CLSI) guidelines (CLSI, 2019). The GP-639 card contains sixteen antimicrobials: penicillin (PEN), tigecycline (TJH), ciprofloxacin (CIP), moxifloxacin (MFX), tetracycline (TCY), vancomycin (VAN), quinopudine-dafoputin (QDA), clindamycin (CLI), linezolid (LNZ), levofloxacin (LVX), erythromycin (ERY), gentamicin (GEN), sulfamethoxazole (SXT), rifampicin (RIF), cefoxitin (FOX), and oxacillin (OXA). *S. aureus* ATCC 29213 is the quality control strain for the assay.

### Virulence genes detection

Several virulence genes, biofilm-forming genes and genes encoding extracellular enzyme of *S. aureus* strains were detected by PCR, including fibronectin-binding proteins A and B (*fnb*A, *fnb*B), clumping factors A and B (*clf*A, *clf*B), fibrinogen binding protein (*fib*), collagen binding adhesin (*cna*), polysaccharide intercellular adhesions A, C, D, and R (*ica*A, *ica*C, *ica*D, *ica*R), Staphylococcal enterotoxins A and B (*sea*, *seb*), Panton–Valentine leucocidin (*pvl*), toxic shock syndrome1 (*tsst*-1), hemolysins a and β (*hla*, *hlb*), staphylokinase (*sak*), hyaluronate lyase (*hys*A), and lipase (*lip*). The primer sequences and PCR amplification reaction conditions were described previously (Zhang et al., 2014; He et al., 2015; Qu, 2019; Wu et al., 2019).

### Statistical analysis

Statistical analysis and the plotting of experimental data were performed using graphPad Prism 8.0 software. The measurement data were tested for normal distribution using K-S normality. Those that conformed to a normal distribution were represented by the mean ± standard deviation. For the data of two groups with homoscedasticity, the independent sample *t*-test was used. For the data of two groups with heteroscedasticity, the *t*-test with a Welch’s correction was used. Measurement data that did not conform to a normal distribution are presented as the median (interquartile range), and data were compared between two groups using the Mann-Whitney *U*-test. Enumeration data were showed as numbers or percentages, and data were compared between groups using the Chi-square or Fisher exact test. *P* < 0.05 was considered statistically significant.

### Ethics statement

The current study was approved by the Ethics Committee of Kunming Children’s Hospital (registration no. 2020-03-134-K01). Written informed consent was exempted, since this retrospective study mainly focused on bacteria and patient intervention was not required.

## Results

### Patient clinical characteristics

Sixty-six children (42 males and 26 females) with invasive *S. aureus* infection were admitted to Kunming Children’s Hospital from January 2019 to December 2021. The patients were 1 day to 14 years of age, of whom 30.30% (20/66) were ≤ 1 year of age. Twenty-seven (40.91%) and 39 (59.09%) patients were infected with MRSA and MSSA, respectively. Community-acquired infections accounted for 83.33% (55/66) of all cases. Patients were frequently admitted to the intensive care unit (28.79%, 19/66), the Department of Orthopedics (18.18%, 12/66), and the Department of Hematology (16.67%, 11/66). Blood was the most common type of clinical specimens, accounting for 62.12% (41/66). The invasive infections were common in patients with leukemia, osteomyelitis and pneumonia. In addition, 33.33% (22/66) of patients have multiple infections and 25.76% (17/66) complicated sepsis. The median length of hospital stay was 24.5 and 23 days in patients with MRSA and MSSA infection, respectively. Six patients had not yet recovered when their parents asked for them to be discharged from the hospital (Table 1).

**TABLE 1:** Demographic and clinical characteristics of patients infected with methicillin-sensitive *Staphylococcus aureus* (MSSA) or methicillin-resistant *Staphylococcus aureus* (MRSA).

Clinical characteristics	All (*n* = 66)	MSSA (*n* = 39)	MRSA (*n* = 27)	*P*-value^a^
**Gender**				
Male	42	26	16	>0.05
Female	24	13	11	>0.05
Age median (interquartile range)	4 (10)	4 (10)	4 (10)	>0.05
**Infection type**				
Community-acquired	55	30	25	>0.05
Hospital-acquired	11	9	2	>0.05
**Departments**				
Intensive care unit	19	10	9	>0.05
Orthopedics	12	8	4	>0.05
Hematology	11	8	3	>0.05
Neonatology	6	5	1	>0.05
Others^b^	18	8	10	>0.05
**Sample type**				
Blood	41	29	12	<0.05
Alveolar lavage fluid	11	5	6	>0.05
Pleural effusion	9	3	6	>0.05
Cerebrospinal fluid	3	1	2	>0.05
Bone marrow	2	1	1	>0.05
**Length of hospital stay**				
Days median (interquartile range)	24 (21.75)	23 (21)	24.5 (25.25)	>0.05
Cases not cured at discharge	6	3	3	>0.05
**Disease spectrum**				
Leukemia	12	9	3	>0.05
Osteomyelitis	8	5	3	>0.05
Pneumonia	7	5	2	>0.05
Bronchial pneumonia	7	2	5	>0.05
Bacteremia	5	3	2	>0.05
Suppurative arthritis	3	1	2	>0.05
Osteomyelitis + sepsis + pneumonia	4	3	1	>0.05
Soft tissue infection + sepsis + pneumonia	3	2	1	>0.05
Pneumonia + sepsis	3	1	2	>0.05
Osteomyelitis + sepsis	2	2	0	>0.05
Soft tissue infection	2	1	1	>0.05
Others^c^	10	5	5	>0.05

^a^Comparison between MRSA and MSSA.

^b^Respiratory Medicine (3 cases); General Internal Medicine (3 cases); Infection (2 cases); Daytime Ward (2 cases); Neurosurgery (2 cases); Nephrology (2 cases); General Surgery (1 case); Digestive Medicine (1 case); Cardiothoracic Surgery (1 case); Neurology (1 case).

^c^One case each: central nervous system infection; soft tissue infection + sepsis; soft tissue infection + purulent meningitis; sepsis; brain abscess + pneumonia; purulent meningitis + sepsis; osteomyelitis + pneumonia + sepsis; infective endocarditis + sepsis + pneumonia; liver abscess + sepsis + pneumonia; pneumonia + sepsis.

### Multilocus sequence typing of strains

**FIGURE 1:**
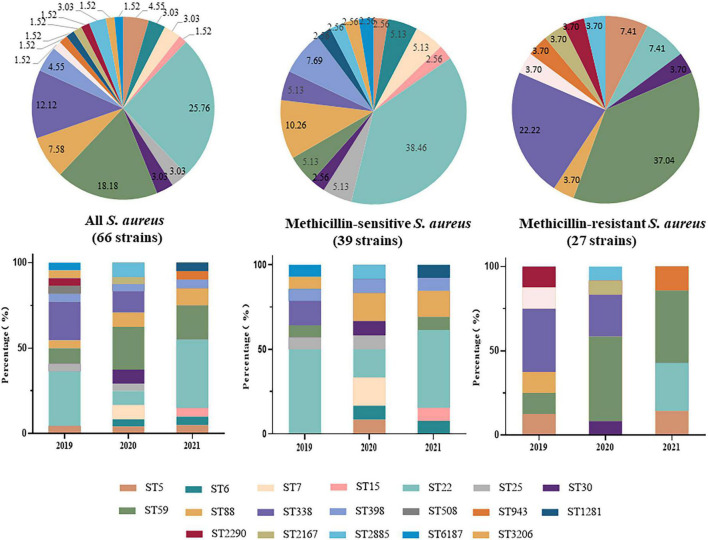
Distribution and changes in Sequence Types (STs) of 66 invasive *Staphylococcus aureus* strains from 2019 to 2021.

Nineteen ST types were identified among the 66 *S. aureus* strains, the most common of which was ST22 (25.76%, 17/66), followed by ST59 (18.18%, 12/66) and ST338 (12.12%, 8/66). ST22 was the dominant ST type among the MSSA strains (15/39, 38.46%), followed by ST88 (4/39, 10.26%). ST59 was the dominant ST type among the MRSA strains (37.04%, 10/27), followed by ST338 (6/27, 22.22%). In 2019 and 2021, the dominant ST type was ST22, while in 2020, it was ST59. After 2019, dominant ST type among MRSA was replaced from ST338 to ST59 (Figure 1).

### *Spa* typing of strains

*Spa* typing failed for three *S. aureus* strains and 31 *spa* types were identified among the remaining 63 *S. aureus* strains. Of these, t309 (24.24%, 16/66) was the dominant *spa* type, followed by t437 (22.73%, 15/66), and the remaining types were only found in one or two strains. Given the MLST results, a strong correlation was found between the dominant *spa* types and ST types, t309 correlated with ST22 (88.24%, 15/17), and t437 correlated with ST59 (75.00%, 9/12) and ST338 (75.00%, 6/8). A correlation was not identified between the remaining *spa* and ST types because of the small number of strains. Thus, the most common *S. aureus* type in this study was ST22-t309 (22.73%, 15/66), followed by ST59-t437 (13.64%, 9/66) and ST338-t437 (9.09%, 6/66). In MSSA strains, the dominant type was ST22-t309 (33.33%, 13/39), while in MRSA strains, the dominant types were ST59-t437 (33.33%, 9/27) and ST338-t437 (14.81%, 4/27) (Table 2).

**TABLE 2:** Molecular characteristics of 66 invasive *Staphylococcus aureus* strains.

*S. aureus*	Sequence types (N,%)	*Spa* types (N)	SCC*mec* types (N)
MRSA (27 strains)	ST59 (10, 37.04)	t437 (9), NT (1)	III (2), IV (7), V (1)
	ST338 (6, 22.22)	t437 (4)	III (3), IV (1)
		t441 (1), t9460 (1)	IV (2)
	ST5 (2, 7.41)	t022 (1), t179 (1)	IV (2)
	ST22 (2, 7.41)	t309 (2)	IV (2)
	ST30 (1, 3.70)	t019 (1)	IV (1)
	ST88 (1, 3.70)	t1764 (1)	IV (1)
	ST508 (1, 3.70)	t015 (1)	IV (1)
	ST943 (1, 3.70)	t091 (1)	IV (1)
	ST2167 (1, 3.70)	t12415 (1)	IV (1)
	ST2290 (1, 3.70)	t688 (1)	IV (1)
	ST2885 (1, 3.70)	t1062 (1)	V (1)
MSSA (39 strains)	ST22 (15, 38.46)	t309 (13), t091 (1), t12442 (1)	
	ST88 (4, 10.26)	t1376 (1), t3155 (1), t12281 (1) NT (1)	
	T398 (3, 7.69)	t011 (1), t034 (1), t571 (1)	
	ST6 (2, 5.13)	t701 (2)	
	ST7 (2, 5.13)	t796 (2)	
	ST25 (2, 5.13)	t227 (1), NT (1)	
	ST59 (2, 5.13)	t528 (1), t136 (1)	
	ST338 (2, 5.13)	t437 (2)	
	ST5 (1, 2.56)	t5353 (1)	
	ST15 (1, 2.56)	t346 (1)	
	ST30 (1, 2.56)	t5580 (1)	
	ST1281 (1, 2.56)	t164 (1)	
	ST2885 (1, 2.56)	t13849 (1)	
	ST3206 (1, 2.56)	t345 (1)	
	ST6187 (1, 2.56)	t309 (1)	

MSSA, methicillin-sensitive Staphylococcus aureus; MRSA, methicillin-resistant Staphylococcus aureus.

### Staphylococcal cassette chromosome mec typing of methicillin-resistant *Staphylococcus aureus* strains

Three SCC*mec* types were detected in MRSA strains, SCC*mec*IV (74.07%, 20/27), SCC*mec*III (18.52%, 5/27) and SCC*mec*V (7.41%, 2/27). MLST, *spa*, and SCC*mec* typing showed that the major MRSA molecular types in present study were ST59-t437-SCC*mec*IV (25.93% 7/27) and ST338-t437-III (11.11%, 3/27) (Table 2).

### Analysis of antimicrobial resistance

**FIGURE 2:**
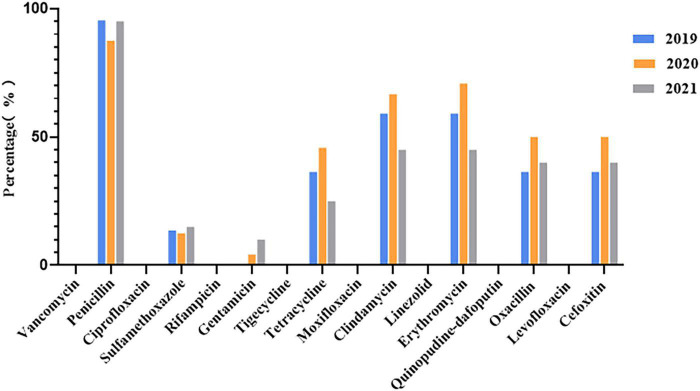
Changes in antimicrobial resistance rates of 66 invasive *Staphylococcus aureus* strains from 2019 to 2021.

**FIGURE 3:**
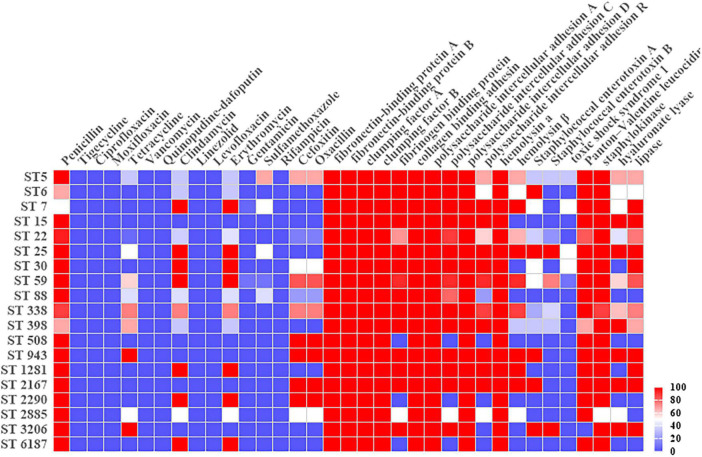
Heatmap of antimicrobial resistance rates and virulence gene carrier rates in different Sequence-Type *Staphylococcus aureus* strains.

VITEK 2 Compact testing showed that 66 *S. aureus* strains were susceptible to TJH, CIP, MFX, VAN, QDA, LNZ, LVX, and RIF, and that the resistance rates to PEN, ERY, CLI, OXA, FOX, TCY, SXT, and GEN were 92.42, 59.09, 57.58, 40.91, 40.91, 34.85, 12.12, and 3.03%, respectively (Table 3). Further analysis showed that the resistance rates to PEN and SXT initially decreased and then increased from 2019 to 2021, a trend that contrasted with the resistance rates to OXA, FOX, ERY, CLI, and TCY (Figure 2). And there were differences in antimicrobial resistance rates among different ST-type strains, for example, ST59 was significantly more resistant to PEN, TCY, ERY, CLI, OXA, and FOX than ST22 and ST338 (Figure 3). In this study, *S. aureus* had 16 different antimicrobial resistance profiles, the most common being resistance to PEN-TCY-ERY-CLI-OXA-FOX (10/66, 15.15%), followed by resistance to PEN only and PEN-ERY-CLI (9/66, 13.64% for each profile) (Table 4). There were some differences in the antimicrobial resistance profiles by year. The most common antimicrobial resistance profiles in 2019 and 2021 were resistance to PEN only and PEN-ERY-CLI, while in 2020 it was resistance to PEN-TCY-ERY-CLI-OXA-FOX (Table 4).

**TABLE 3:** Antimicrobial resistance profiles of 66 invasive *Staphylococcus aureus* strains.

Antimicrobial	Resistant N (%)	Sensitive N (%)	Intermediate N (%)
Penicillin	61 (94.42)	5 (7.58)	0
Tigecycline	0	66 (100.00)	0
Ciprofloxacin	0	65 (98.48)	1 (1.52)
Moxifloxacin	0	66 (100.00)	0
Tetracycline	23 (34.85)	43 (65.15)	0
Vancomycin	0	66 (100.00)	0
Quinopudine-dafoputin	0	66 (100.00)	0
Clindamycin	38 (57.58)	28 (42.42)	0
Linezolid	0	66 (100.00)	0
Levofloxacin	0	66 (100.00)	0
Erythromycin	39 (59.09)	27 (40.91)	0
Gentamicin	2 (3.03)	60 (90.91)	4 (6.06)
Sulfamethoxazole	8 (12.12)	58 (87.88)	0
Rifampicin	0	66 (100.00)	0
Cefoxitin	27 (40.91)	39 (59.09)	0
Oxacillin	27 (40.91)	39 (59.09)	0

**TABLE 4:** Antimicrobial resistance profiles of invasive *Staphylococcus aureus* strains from 2019 to 2021.

Antimicrobial resistance profiles	All (66 strains) N (%)	2019 (22 Strains) N (%)	2020 (24 Strains) N (%)	2021 (20 Strains) N (%)
All sensitive	3 (4.55)	0	2 (8.33)	1 (5.00)
PEN	9 (13.64)	3 (13.64)	1 (4.17)	5(25.00)
PEN-TCY	5 (7.58)	3 (13.64)	1 (4.17)	1 (5.00)
PEN-ERY	1 (1.52)	0	1 (4.17)	0
ERY-CLI	2 (3.03)	1 (4.55)	1 (4.17)	0
PEN-SXT	1 (1.52)	0	1 (4.17)	0
PEN-ERY-CLI	9 (13.64)	4 (18.18)	1 (4.17)	4 (20.00)
PEN-OXA-FOX	5 (7.58)	1 (4.55)	2 (8.33)	2 (10.00)
PEN-TCY-ERY-CLI	4 (6.06)	2 (10.00)	2 (8.33)	0
PEN-TCY-OXA-FOX	3 (4.55)	1 (4.55)	0	2 (10.00)
PEN-ERY-CLI-SXT	3 (4.55)	1 (4.55)	1 (4.17)	1 (5.00)
PEN-SXT-OXA-FOX	1 (1.52)	1 (4.55)	0	0
PEN-ERY-CLI-OXA-FOX	7 (10.61)	2 (10.00)	2 (8.33)	3 (15.00)
PEN-TCY-ERY-CLI-OXA-FOX	10 (15.15)	2 (10.00)	8 (33.33)	0
PEN-ERY-CLI-SXT-OXA-FOX	1 (1.52)	1 (4.55)	0	0
PEN-TCY-ERY-CLI-GEN-SXT	2 (3.03)	0	1 (4.17)	1 (5.00)

PEN, penicillin; TCY, tetracycline; ERY, erythromycin; CLI, clindamycin; SXT, sulfamethoxazole; OXA, oxacillin; FOX, cefoxitin; GEN, gentamicin.

### Detection of virulence genes

The carrier rates for common *S. aureus* virulence genes, *ica*A, *ica*D, *fnb*A, *fnb*B, *clf*A, *clf*B, *hla*, and *cna*, were 100.00%. *sak*, *pvl*, *ica*C, *ica*R, *fib*, *lip*, *hlb*, and *hysA* had carrier rates of 96.97, 92.42, 87.88, 69.70, 84.85, 62.12, 56.06, and 50%, respectively, while *sea*, *seb*, and *tsst-1* had carrier rates of 37.87, 30.30, and 7.58%, respectively. The distribution of virulence genes in MRSA and MSSA strains did not differ significantly, but there were some differences in ST type strains, particularly for *ica*R, *hlb*, *sea*, *seb*, *hys*A, and *lip* (Table 5 and Figure 3).

**TABLE 5:** Carrying rates of virulence genes in 66 invasive *Staphylococcus aureus* strains.

Gene	All (66 strains) N (%)	MSSA (39 strains) N (%)	MRSA (27 strains) N (%)	*P*-value^a^
*fnb*A	66 (100.00)	39 (100.00)	27 (100.00)	N
*fnb*B	66 (100.00)	39 (100.00)	27 (100.00)	N
*clf*A	66 (100.00)	39 (100.00)	27 (100.00)	N
*clf*B	66 (100.00)	39 (100.00)	27 (100.00)	N
*fib*	56 (84.85)	33 (84.62)	23 (85.19)	> 0.05
*cna*	66 (100.00)	39 (100.00)	27 (100.00)	N
*ica*A	66 (100.00)	39 (100.00)	27 (100.00)	N
icaC	58 (87.88)	35 (89.74)	23 (85.19)	> 0.05
*ica*D	66 (100.00)	39 (100.00)	27 (100.00)	N
*ica*R	46 (69.70)	24 (61.54)	22 (81.48)	> 0.05
*hla*	66 (100.00)	39 (100.00)	27 (100.00)	N
*hlb*	41 (62.12)	22 (56.41)	19 (70.37)	> 0.05
*sea*	25 (37.87)	13 (33.33)	12 (44.44)	> 0.05
*seb*	20 (30.30)	9 (23.08)	11 (40.74)	> 0.05
*tst*-1	5 (7.58)	3 (7.69)	2 (7.41)	> 0.05
*pvl*	61 (92.42)	34 (87.18)	27 (100.00)	> 0.05
*sak*	64 (96.97)	38 (97.44)	26 (96.30)	> 0.05
*hys*A	33 (50.00)	17 (43.59)	16 (59.26)	> 0.05
*lip*	45 (68.18)	27 (69.23)	18 (66.67)	> 0.05

MSSA, methicillin-sensitive Staphylococcus aureus; MRSA, methicillin-resistant Staphylococcus aureus.

^a^Comparison between MRSA and MSSA.

fnbA and fnbB, fibronectin-binding proteins A and B; clfA and clfB, clumping factors A and B; fib, fibrinogen binding protein; cna, collagen binding adhesin; icaA, icaC, icaD and icaR, polysaccharide intercellular adhesions A, C, D and R; sea and seb, Staphylococcal enterotoxins A and B; pvl, Panton–Valentine leucocidin; tsst-1, toxic shock syndrome 1; hla and hlb, hemolysins a and β; sak, staphylokinase; hysA, hyaluronate lyase gene; lip, lipase.

### Comparison of the characteristics in the dominant strains ST22-t309, ST338-t437, and ST59-t437

#### Antimicrobial resistance rates and profiles

Except for PEN, ST338-t437 strains had higher resistance rates to CLI, ERY, OXA, and FOX than ST22-t309 strains. ST59-t437 strains were resistant to PEN, ERY, CLI, OXA, and FOX, with a TCY resistance rate of 66.67%. MRSA was found in only 13.33% of ST22-t309 strains, but in 66.67% of ST338-t437 strains and 100.00% of ST59-t437 strains, respectively (Table 6). Resistance to PEN-ERY-CLI and PEN alone was the most common resistance profile of the ST22-t309 strains, while resistance to PEN-TCY-ERY-CLI-OXA-FOX was the most common resistance profile of the ST338-t437 and ST59-t437 strains (Table 7).

**TABLE 6:** Antimicrobial resistance rates of ST22-t309, ST338-t437, and ST59-t437.

Antimicrobial	ST22-t309 (15 strains) N (%)	ST338-t309 (6 strains) N (%)	ST59-t437 (9 strains) N (%)
Penicillin	14 (93.33)	5 (83.33)	9 (100)
Tigecycline	0	0	0
Ciprofloxacin	0	0	0
Moxifloxacin	0	0	0
Tetracycline	0	0	6 (66.67)
Vancomycin	0	0	0
Quinopudine-dafoputin	0	0	0
Clindamycin	6 (40.00)	4 (66.67)	9 (100)
Linezolid	0	0	0
Levofloxacin	0	0	0
Erythromycin	7 (46.67)	4 (66.67)	9 (100)
Gentamicin	0	0	0
Sulfamethoxazole	0	0	0
Rifampicin	0	0	0
Cefoxitin	2 (13.33)	4 (66.67)	9 (100)
Oxacillin	2 (13.33)	4 (66.67)	9 (100)

**TABLE 7:** Antimicrobial resistance profiles of ST22-t309, ST338-t437, and ST59-t437.

Antimicrobial resistance profiles	ST22-t309 (15 strains) N (%)	ST338-t309 (6 strains) N (%)	ST59-t437 (9 strains) N (%)
All sensitive	1 (6.67)	0	0
PEN	5 (33.33)	0	0
PEN-ERY	1 (6.67)	0	0
PEN-CLI	0	1 (16.67)	0
PEN-ERY-CLI	6 (40.00)	0	0
PEN-OXA-FOX	2 (13.33)	1 (16.67)	0
PEN-TCY-ERY-CLI	0	1 (16.67)	0
PEN-TCY-OXA-FOX	0	1 (16.67)	0
PEN-ERY-CLI-OXA-FOX	0	0	3 (33.33)
PEN-TCY-ERY-CLI-OXA-FOX	0	2 (33.33)	6 (66.67)

PEN, penicillin; ERY, erythromycin; CLI, clindamycin; OXA, oxacillin; FOX, cefoxitin; TCY, tetracycline.

#### Virulence genes

In the three dominant strains, *fnb*A, *fnb*B, *cna*, *ica*A, *ica*D, and *hla* had 100.00% carrier rates. While the carrier rates of *fib*, *ica*C, *hlb*, and *pvl* in the ST59-t437 and ST338-t437 strains were 100.00%, which were significantly greater than the carrier rates in the ST22-t309 strains. The carrier rates of *ica*R, *seb* and *hys*A were highest in the ST59-t437 strains, followed by the ST338-t437 and ST22-t309 strains (Table 8).

**TABLE 8:** Carrying rates of virulence genes in ST22-t309, ST338-t437, and ST59-t437.

Gene	ST22-t309 (15 strains) N (%)	ST338-t309 (6 strains) N (%)	ST59-t437 (9 strains) N (%)
*fnb*A	15 (100.00)	6 (100.00)	9 (100.00)
*fnb*B	15 (100.00)	6 (100.00)	9 (100.00)
*clf*A	15 (100.00)	6 (100.00)	9 (100.00)
*clf*B	15 (100.00)	6 (100.00)	9 (100.00)
*fib*	10 (66.67)	6 (100.00)	9 (100.00)
*cna*	15 (100.00)	6 (100.00)	9 (100.00)
*ica*A	15 (100.00)	6 (100.00)	9 (100.00)
*ica*C	13 (86.67)	6 (100.00)	9 (100.00)
*ica*D	15 (100.00)	6 (100.00)	9 (100.00)
*ica*R	8 (53.33)	5 (83.33)	9 (100.00)
*hla*	15 (100.00)	6 (100.00)	9 (100.00)
*hlb*	10 (66.67)	6 (100.00)	9 (100.00)
*sea*	5 (33.33)	1 (16.67)	5 (55.56)
*seb*	3 (20.00)	3 (50.00)	8 (88.89)
*tst*-1	0	0	1 (11.11)
*pvl*	12 (80.00)	6 (100.00)	9 (100.00)
*sak*	15 (100.00)	5 (83.33)	9 (100.00)
*hys*A	6 (40.00)	3 (50.00)	7 (77.78)
*lip*	11 (73.33)	4 (66.67)	8 (88.89)

fnbA and fnbB, fibronectin-binding proteins A and B; clfA and clfB, clumping factors A and B; fib, fibrinogen binding protein; cna, collagen binding adhesin; icaA, icaC, icaD and icaR, polysaccharide intercellular adhesions A, C, D and R; sea and seb, Staphylococcal enterotoxins A and B; pvl, Panton–Valentine leucocidin; tsst-1, toxic shock syndrome 1; hla and hlb, hemolysins a and β; sak, staphylokinase; hysA, hyaluronate lyase gene; lip, lipase.

## Discussion

This study collected 66 invasive *S. aureus* strains at Kunming Children’s Hospital from 2019 to 2021 and analyzed them for molecular typing, antimicrobial resistance, and virulence genes. The findings revealed a unique prevalence characteristic and change in invasive *S. aureus* strains found among children in this region, which differ from those reported from other countries and regions of China. Moreover, the molecular characteristics of prevalent strains in Kunming have changed significantly since the COVID-19 epidemic.

In this study, 66 children with invasive *S. aureus* infection were detected in three years (22, 24, and 20 in 2019, 2020, and 2021, respectively), showing little change in the annual number of cases. This suggests that the increased use of personal protection during the COVID-19 epidemic did not significantly reduce the incidence of this invasive disease, which is consistent with the findings of McNeil et al. (2021). Some studies have found that family members and household environment play a key role in *S. aureus* transmission (Mork et al., 2018; Mork et al., 2020). Therefore, increased time spent indoors and among household contacts may have contributed to the relatively stable incidence of invasive *S. aureus* infection during COVID-19 epidemic. The age of the children varied widely from 1 day to 14 years, however, 30.30% of the patients were < 1 year of age. In addition, invasive *S. aureus* infection was frequently seen among children with leukemia in present study. These findings suggest that such infections are likely to occur in infants and young children with underdeveloped immune systems or immunodeficiency. The patients in this study were primarily treated in the ICU and in the departments of orthopedics, and hematology, which is consistent with the characteristics of the diseases covered by these departments. Patients in the ICU and the department of orthopedics often receive invasive therapies such as surgery, invasive ventilation, gastric tube placement, and tracheal intubation or incision. Many children in the department of hematology are immunodeficient or are on tumor chemotherapy drugs so often have compromised immunity and may be more susceptible to invasive infections.

Data from the China Antimicrobial Surveillance Network^3^ indicates that the MRSA rate in China has dropped from 31.40% in 2019 to 30.00% in 2021, showing a decreasing annual trend. In this study, however, the average detection rate of MRSA was 40.91% from 2019 to 2021. And the detection rate of MRSA was also higher than the national average each year, which may be related to different regions or infection types. In addition, the MSSA rate (59.09%) in this study was much higher than that observed for MRSA (40.91%). This suggests that MSSA is the most common cause of invasive *S. aureus* infection among children in Kunming. While MRSA has gained considerable attention for its multi-drug resistance since its emergence in the 1980s, MSSA has been largely ignored, especially regarding invasive *S. aureus* infection. Some studies found that MSSA was more likely to cause bacteremia, endocarditis, and sepsis than MRSA and that there may be a “role reversal” between these two types of drug-resistant bacteria (Diep et al., 2011; Koeck et al., 2019). In China, current *S. aureus-*related infections are primarily caused by MSSA, so this bacterium should not be ignored in favor of focusing solely on MRSA. Resistance to TJH, VAN, QDA, LNZ, and RIF was not found in this study, so these antibiotics could be used as a clinical treatment for relevant infections.

In this study, the 66 *S. aureus* strains belonged to 19 ST types and 31 *spa* types, indicating that *S. aureus* strains in Kunming have a diverse genetic background. ST22, ST59, and ST338 were the top three ST types, accounting for up to 56.06% of all strains. ST22 and ST88 were the dominant MSSA ST types. ST59 and ST338 were the dominant MRSA ST types. Qiao et al. (2014) reported that ST88, ST25, and ST7 were the most common ST types of strains responsible for invasive CA-MSSA infection among Chinese children, and no ST22 strains were found, which differs substantially from the results of the current study. ST22, a highly transmissible strain originating from Europe, has caused several major outbreaks in Europe, India, Gaza Strip, and Singapore, and has become one of the main MRSA ST types worldwide (Tinelli et al., 2009; Manoharan et al., 2012; Hsu et al., 2015; Chang et al., 2018; Htun et al., 2018). In 2013, Li et al. (2013) first identified two ST22 strains in Shanghai, both of which belonged to MSSA. The detection rate of ST22 was low at the time, which may explain why this study’s results differed from Qiao’s. At present, ST22 becomes the dominant MSSA strain in several important Chinese cities such as Beijing, Chongqing, and Urumqi (Yang et al., 2017; Xiao et al., 2019; Yuan et al., 2019). According to the findings of this study, ST22 may have also become the predominant ST type of MSSA strains in Kunming. Unfortunately, current researches on invasive *S. aureus* infections in China have primarily focused on MRSA, and there have been no reports on the predominance of ST22. Many studies have found that ST59 is the predominant ST type among MRSA strains of invasive *S. aureus* infections in China (Qiao et al., 2013; Li et al., 2015), supporting the results of this study.

Previous studies have shown that virulence factors play an important role in severe *S. aureus* infections, and the types and numbers of *S. aureus* strains carrying virulence genes are closely related to their invasiveness and pathogenicity (Loughman et al., 2009; Jenkins et al., 2015). In this study, all strains carried the microbial surface components recognizing adhesive matrix molecules (MSCRAMMs) genes, *fnb*A, *fnb*B, *clf*A, *clf*B, and *fib*. The proteins encoded by these genes play an important role in pathogen recognition and adhesion of host cells and extracellular matrix proteins, which are the first steps in disease development (Foster et al., 2014). In addition, the carrier rates of the key biofilm-forming genes, *ica*A, *ica*C, *ica*D, and *ica*R, were high in this study, suggesting that the strains were not only highly virulent but also had a strong ability to form biofilms. Biofilms formed by bacteria not only promote their survival in unfavorable environments but also allow them to acquire drug resistance (Omidi et al., 2020). This may explain the ability of these strains to survive and cause disease during the COVID-19 epidemic. Analysis of several other common *S. aureus* virulence genes showed that the hemolysin genes, *hla* and *hlb*, had the highest carrier rates (62.12–100.00%). These genes encode hemolysin, a factor that destroys multiple host cell types, promotes inflammation and causes *S. aureus* pneumonia and sepsis in clinical practice. It is also worth noting that the *pvl* gene carrier rate in this study was 92.42%. Several studies have shown that *pvl* has a high carrier rate in ST22 strains (Yang et al., 2017; Yuan et al., 2019), and in our study, it was as high as 82.35% in ST22 strains. Previous studies have shown the association between PVL and severe invasive infections (Yonezawa et al., 2015; Rajova et al., 2016). Furthermore, PVL is shown to play an important role in the development of pneumonia, and *pvl* gene expression level is positively correlated with pneumonia severity, which may explain why almost half of the patients in this study developed this complication. The three extracellular enzyme genes, *sak*, *hysA*, and *lip*, also had a higher carrying rates in this study (96.97, 50.00, and 68.18%, respectively). Therefore, invasive *S. aureus* infection results from the combination of multiple *S. aureus* toxins and extracellular enzymes. Further analysis showed no apparent differences in the carrier rates of these genes between MSSA and MRSA strains, suggesting that the virulence of MSSA and MRSA strains responsible for invasive infection in children is comparable.

The molecular types of *S. aureus* by geographical region vary over time, a finding also demonstrated in this study. The dominant *S. aureus* strain responsible for invasive infection in children from Kunming was ST22-t309 in 2019 and ST59-t437 in 2020. However, the dominant *S. aureus* strain returned to ST22-t309 in 2021. At the same time, dominant MRSA strain was replaced from ST338-t309 to ST59-t437. Thus, the dominant *S. aureus* and MRSA strains responsible for invasive infection in this region have changed over time. This could have resulted from (i) natural changes in prevalent strains or (ii) To prevent COVID-19 infection and transmission since the end of 2019, various prevention and control measures were taken. Local citizens were required to wear a mask, home quarantine was enforced, personal hygiene was stressed, and individual lifestyles changed dramatically. These alterations may have driven changes in the prevalent *S. aureus* strain type. Additional analysis of the antimicrobial resistance and virulence of the three dominant types showed that ST59-t437 had a stronger resistance to common antibiotics, a broader antimicrobial resistance profile, and higher carrying rates of various virulence genes than ST22-t309 and ST338-t437. These characteristics may have contributed to the survival and transmission of the ST59-t437 strain during the COVID-19 epidemic, especially when it was worst in 2020, allowing ST59-t437 to become the absolute dominant strain. In contrast, before the COVID-19 epidemic in 2019, and once the COVID-19 epidemic in China had been obviously mitigated and personal protection was reduced in 2021, ST22-t309 became the major prevalent strain.

There are limitations to this study which should be acknowledged. Foremost, our results are from a single center, which may affect the representativeness of the samples. In addition, the sample size was small and type II errors could not be overcome. Future multi-center, large-sample studies will be required to confirm the findings of the present study.

In summary, our results showed that the annual number of invasive *S. aureus* strains isolated from children in Kunming remained stable during 2019–2021. The dominant molecular types of strains were ST22-t309 and ST59-t437, and with diverse genetic backgrounds. The molecular characteristics and antimicrobial resistance profiles of prevalent *S. aureus* strains changed significantly during the COVID-19 epidemic. Thus, COVID-19 prevention and control should be supplemented by surveillance of common clinical pathogens, particularly vigilance against the prevalence of multidrug-resistant and high-virulence strains.

## Data availability statement

The raw data supporting the conclusions of this article will be made available by the authors, without undue reservation.

## Author contributions

MM and TD conceived and designed the experiments. YL and LT contributed to the molecular experiment and analyses of the data. XL, JL, and HW contributed to the identification of strains and antibiotic-susceptibility testing. HJ, JD, and DH contributed to collection of strains and data. All authors contributed to drafting the manuscript, critically revising the manuscript, and approved the final version of the manuscript.

## Footnotes

1
https://pubmlst.org/

2
http://spaserver.ridom.de

3
www.chinets.com
